# Multicentric performance analysis of HCV quantification assays and its potential relevance for HCV treatment

**DOI:** 10.1007/s00430-015-0443-9

**Published:** 2015-12-14

**Authors:** F. Wiesmann, G. Naeth, A. Berger, H. H. Hirsch, S. Regenass, R. S. Ross, C. Sarrazin, H. Wedemeyer, H. Knechten, P. Braun

**Affiliations:** PZB Aachen, HIV&Hepatitis Research Group, PZB Aachen Blondelstr. 9, Aachen, Germany; University of Frankfurt, Frankfurt, Germany; Institute of Medical Microbiology, University of Basel, Basel, Switzerland; Clinic for Immunology, University Hospital Zurich, Zurich, Switzerland; University Hospital - National Reference Centre for HCV, University of Duisburg-Essen, Essen, Germany; Institute of Virology, MHH Hannover, Hannover, Germany

**Keywords:** HCV, Viral load, RealTi*m*e, COBAS Taqman, Assay variation

## Abstract

An accurate quantification of low viremic HCV RNA plasma samples has gained importance since the approval of direct acting antivirals and since only one single measurement predicts the necessity of a prolonged or shortened therapy. As reported previously, HCV quantification assays such as Abbott RealTi*m*e HCV and Roche COBAS AmpliPrep/COBAS TaqMan HCV version 2 (CTM v2) may vary in sensitivity and precision particularly in low-level viremia. Importantly, substantial variations were previously demonstrated between some of these assays compared to the Roche High Pure System/COBAS TaqMan assay (HPS) reference assay, which was used to establish the clinical decision points in clinical studies. In this study, the reproducibility of assay performances across several laboratories was assessed by analysing quantification results generated by six independent laboratories (3× RealTi*m*e, 3× CTM v2) in comparison with one HPS reference laboratory. The 4th WHO Standard was diluted to 100, 25 and 10 IU/ml, and aliquots were tested in triplicates in 5 independent runs by each assay in the different laboratories to assess assay precision and detection rates. In a second approach, 2 clinical samples (GT 1a & GT 1b) were diluted to 100 and 25 IU/ml and tested as described above. While the result range for WHO 100 IU/ml replicates across all laboratories was similar in this analysis, the CVs of each laboratory ranged from 19.3 to 25.6 % for RealTi*m*e laboratories and were lower than CVs of CTM v2 laboratories with a range of 26.1–47.3 %, respectively, and also in comparison with the CV of the HPS reference laboratory (34.9 %). At WHO standard dilution of 25 IU/ml, 24 replicates were quantified by RealTi*m*e compared to 8 replicates with CTM v2. Results of clinical samples again revealed a higher variation of CTM v2 results as compared to RealTi*m*e values. (CVs at 100 IU/ml: RealTi*m*e: 13.1–21.0 % and CTM v2: 15.0–32.3 %; CVs at 25 IU/ml: RealTi*m*e 17.6–34.9 % and CTM v2 28.2–54.9 %). These findings confirm the superior precision of RealTi*m*e versus CTM v2 at low-level viremia even across different laboratories including the new clinical decision point at 25 IU/ml. A highly precise monitoring of HCV viral load during therapy will remain crucial for patient management with regard to futility rules, therapy efficacy and SVR.

## Introduction

To date, the most widely used HCV RNA quantification assays are the Abbott RealTi*m*e HCV assay (RealTi*m*e) and the Roche COBAS AmpliPrep/COBAS TaqMan HCV test version 2 (CTM v2). They were calibrated with historical WHO standards (2nd) and may vary in sensitivity and precision depending on genotypes and particularly in the setting of low-level viremia, as previously reported [1–3]. A major role in DAA therapy (direct acting antivirals) was assigned to HCV RNA monitoring not only for investigating initial viral load but also for assessing patient adherence, sustained virological response (SVR; defined as HCV RNA < 25 IU/ml in DAA registration trials) and to follow stopping rules at 25 IU/ml for the compounds *Simeprevir* and *Daclatasvir* or even HCV RNA detectability or undetectability at certain points of time during therapy [4–6, 8, 9]. Viral loads below or above these thresholds are intended to guide therapy decision, in particular at the end of treatment. The clinical decision points were implemented using the manual Roche High Pure System/COBAS TaqMan assay (HPS) which, however, is rarely used in clinical routine.

The comparison of six commercially available HCV RNA assays (one laboratory per assay) revealed a substantial variety in assay precision at low viremic levels in particular at 25 and 100 IU/ml [7]. Additionally, substantial differences in quantification were also described between the HPS reference assay, which was used to establish the clinical decision points in clinical studies, and some assay comparators in this recent analysis [7].

In order to confirm the results of this initial HCV low-level viremia (LLV) study that used a one-laboratory-per-assay approach and in order to assess laboratory-specific biases, the present study focussed on the assessment of precision and detection rates of RealTi*m*e and CTM v2 results obtained by three independent laboratories each (multiple laboratories per assay) and using diluted 4th HCV WHO Standard samples as well as low viremic clinical samples near detection limits of chronically HCV-infected patients.

## Materials and methods

Dilutions were prepared from the 4th WHO standard (NIBSC code: 06/102, genotype 1a) to achieve nominal concentrations of 100, 25 and 10 IU/ml using HCV-negative BaseMatrix (Seracare Life Sciences; HCV negativity was confirmed by using the Abbott RealTi*m*e HCV assay). For each participating laboratory, fifteen aliquots of each dilution level were prepared which were treated identically and underwent the same freeze-thawing cycles. Samples were transported on dry ice to the participating laboratories and successfully delivered in a frozen state.

Seven different laboratories participated in this study with 3 laboratories using RealTi*m*e (PZB Aachen, Germany/University of Essen, Germany/University of Basel, Switzerland), 3 laboratories using CTM v2 (University of Frankfurt, Germany/MHH Hannover, Germany/University Hospital Zurich, Switzerland) and 1 laboratory using HPS (University of Frankfurt, Germany). Assay characteristics as provided by the manufacturer are listed in Table 1.

**Table 1:** Assay characteristics as provided by manufacturers

	CTM v2	HPS	RealTi*m*e
Manufacturer	Roche	Roche	Abbott
Reference standard	2nd WHO	2nd WHO	2nd WHO
Input volume	0.5 ml	0.5 ml	0.5 ml
Target region	5′UTR	5′UTR	5′UTR
LLOQ	15 IU/ml	25 IU/ml	12 IU/ml
LOD	15 IU/ml	~9.3–16.1 IU/ml	12 IU/ml

*LLOQ* lower limit quantification, *LOD* limit of detection

In each laboratory, three aliquots of each dilution were tested in five independent runs, respectively. Medians, coefficients of variation (CV) and their confidence intervals were determined for 100 IU/ml dilutions and detection rates for 25 and 10 IU/ml dilutions.

In a second approach, two clinical samples (one genotype 1a and one genotype 1b sample) were diluted in BaseMatrix to concentrations of 100 and 25 IU/ml and were transported to all participating centres. On each system, three replicates of each concentration were tested in five independent runs (15 aliquots in total), respectively, and CV values, confidence intervals as well as medians were determined. The initial HCV RNA concentrations of the clinical samples had been determined with the Abbott RealTi*m*e HCV system prior to dilution.

CVs were only calculated if at least 2 of 3 replicates in each of at least 4 of 5 runs per sample dilution were quantified.

## Results

### WHO standard dilutions

#### Assay result variation for WHO standard replicates

**Fig. 1:**
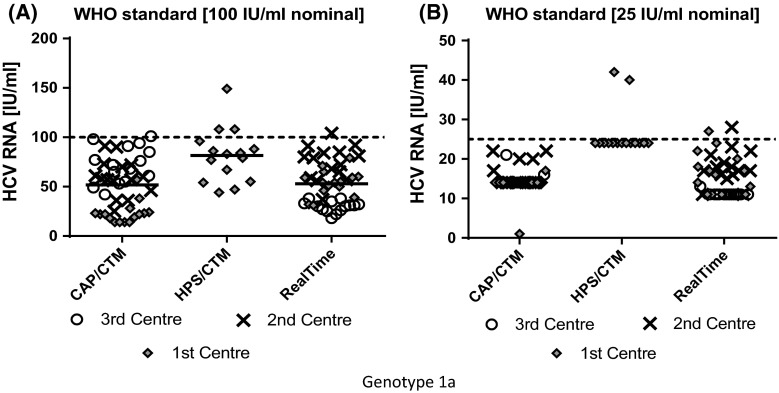
Total WHO standard variation analysis was performed in triplicates in 5 independent runs (3 × 5 replicates) for each participating centre, as indicated by different marks. **a** WHO standard replicates with a nominal concentration of 100 IU/ml (*dotted line*). **b** WHO standard replicates with a nominal concentration of 25 IU/ml

The overall testing results of WHO standard replicates at nominal concentrations of 100 and 25 IU/ml are shown in Fig. 1a, b. Results of the different laboratories are differentiated by blue, green and red colour. While the overall result range across all laboratories for 100 IU/ml replicates was similar for CTM v2 and RealTi*m*e in this analysis (Fig. 1: all colours), the CVs of the individual laboratories were lower across RealTi*m*e results with a range of 19.3–25.6 % compared to CVs of CTM v2 laboratories with a range of 26.1–47.3 % and the HPS laboratory with a CV of 34.9 % (Table 2). As shown in Table 2, overall median values across all replicates at 100 IU/ml and across all laboratories were similar for CTM v2 (56 IU/ml; range 22–72 IU/ml per laboratory) and RealTi*m*e (54 IU/ml; range 31–79 IU/ml per laboratory) and differed moderately from HPS median in this analysis (83 IU/ml) (Table 2). As shown in Fig. 1b, three-times more replicates at 25 IU/ml were quantified with RealTi*m*e (24) than with CTM v2 (8), with one CTM v2 centre (blue colour) and one RealTi*m*e centre (red colour) quantifying only one replicate each. In total, two replicates were quantified above 25 IU/ml in the HPS centre compared to two replicates by RealTi*m*e and none by CTM v2. Due to the low number of quantified replicates, a calculation of CV values at 25 IU/ml was not possible.

**Table 2:** Median and CV data for the WHO dilution at 100 IU/ml nominal concentration

WHO 100 IU/ml	Median per laboratory (IU/ml)*			Overall median (IU/ml)	CV % per laboratory* (95 % Confidence interval)		
CTM v2	22	58	72	56	26.1 (18.8–42.8)	34.1 (24.3–57.6)	47.3 (30.8–104.6)
HPS	83			83	34.9 (24.9–59.2)		
RealTi*m*e	31	54	79	54	19.3 (13.9–31.9)	23.4 (16.9–38.1)	25.6 (18.5–42.0)

* All values in increasing order independent of laboratory

#### Analysis of assay detection rates for WHO standard replicates

Furthermore, the ability of each laboratory and each assay to detect HCV RNA in 15 WHO replicates at nominal concentrations of 25 and 10 IU/ml, respectively, was analysed (Table 3).

**Table 3:** Detection rates for 4th WHO replicates with nominal concentrations of 25 and 10 IU/ml

Assay	25 IU/ml nominal concentration			10 IU/ml nominal concentration		
	Not det.	Det. < LOQ	≥LOQ	Not det.	Det. < LOQ	≥LOQ
HPS (total)	0/15 (0 %)	13/15 (87 %)	2/15 (13 %)	3/15 (20 %)	12/15 (80 %)	0/15 (0 %)
Centre #1	0	13	2	3	12	0
CTM v2	1/45 (2 %)	36/45 (80 %)	8/45 (18 %)	10/45 (22 %)	34/45 (76 %)	1/45 (2 %)
Centre #2	1	13	1	6	9	0
Centre #3	0	10	5	1	13	1
Centre #4	0	13	2	3	12	0
RealTi*m*e	0/44* (0 %)	20/44* (45 %)	24/44* (55 %)	3/45 (7 %)	40/45 (89 %)	2/45 (4 %)
Centre #5	0	5	10	2	13	0
Centre #6	0	2	13	1	12	2
Centre #7	0	13	1	0	15	0

* One replicate missing due to technical issues

Overall, in nearly all 25 IU/ml replicates, HCV RNA was detected by CTM v2, HPS and RealTi*m*e. However, the amount of quantified replicates differed between CTM v2 (18 %) and RealTi*m*e (55 %). HPS, which has a limit of quantification of 25 IU/ml, quantified 13 % of 15 replicates. For replicates at 10 IU/ml, 22 % were not detected by CTM v2 centres compared to 7 % by RealTi*m*e laboratories. The HPS reference centre did not detect 20 % of replicates. Overall detection rates were 97 % for RealTi*m*e, 88 % for CTM v2 and 90 % for HPS.

#### Analysis of assay precision and quantification of replicates of clinical samples with genotypes 1a and 1b

**Fig. 2:**
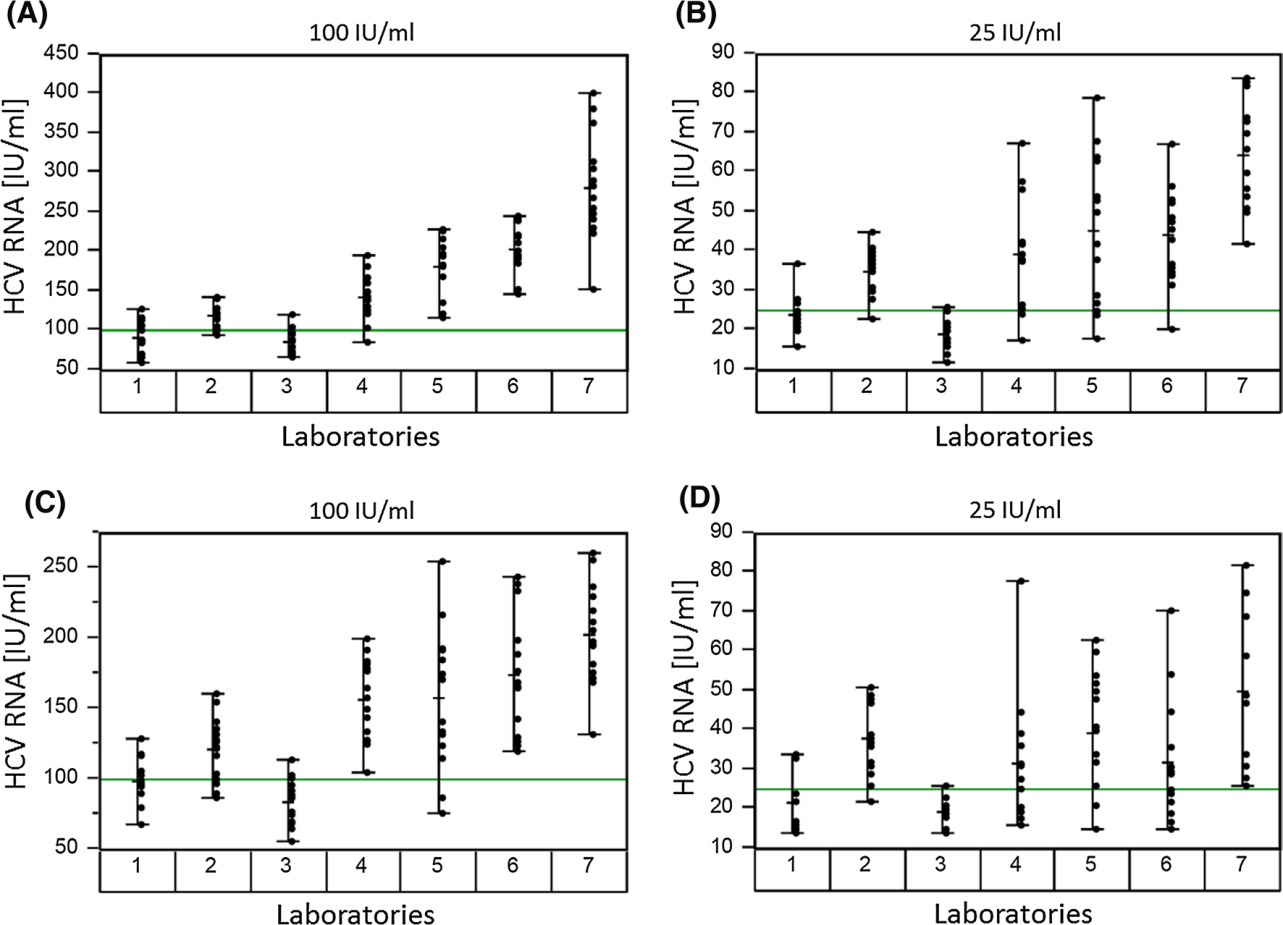
Variability charts of 15 replicates for each of the clinical genotype 1a sample (**a**, **b**) and genotype 1b sample (**c**, **d**) at nominal concentrations of 100 and 25 IU/ml, respectively. Numbers on the *x*-axis represent the different laboratories (1–3 represent RealTi*m*e centres, 4–6 represent CTM v2 centres, 7 represents the HPS reference centre)

The overall assay variations in clinical genotype 1 samples at nominal concentration of 100 and 25 IU/ml are illustrated in Fig. 2. As already shown for WHO standard replicates, this analysis confirmed differences in precision across the evaluated assays independent of the participating centres (Fig. 2). Results from clinical samples revealed higher precision and lower CVs for RealTi*m*e as compared to CTM v2 (Table 4). The calculated CVs obtained across RealTi*m*e laboratories ranged from 13.1 to 21.0 % for replicates at 100 IU/ml and from 17.6 to 34.9 % for those at 25 IU/ml. In comparison, the calculated CVs obtained across CTM v2 laboratories ranged from 15.0 to 32.3 % at 100 IU/ml and were particularly higher for replicates at 25 IU/ml with CVs of 28.2–54.9 %. The CVs for HPS were 17.7–23.6 % at 100 IU/ml and 22.8–42.5 % at 25 IU/ml (Table 4).

**Table 4:** Median and CV data for clinical genotype 1a and 1b samples

Genotype 1a							
	Median per laboratory (IU/ml)*			Overall median (IU/ml)	CV % per laboratory* (95 % Confidence interval)		
GT1a 100 IU/ml							
CTM v2	141	194	202	184	15.0 (10.9–23.9)	21.7 (15.7–35.2)	23.4 (16.9–38.2)
HPS	269			269	23.6 (17.1–38.5)		
RealTi*m*e	84	87	119	98	13.1 (9.4–21.3)	18.3 (13.2–30.1)	21.0 (15.2–34.0)
GT1a 25 IU/ml							
CTM v2	38	42	46	39	28.2 (20.2–46.6)	37.5 (26.0–67.9)	42.2 (29.4–76.0)
HPS	66			66	22.8 (16.5–37.1)		
RealTi*m*e	20	23	36	24	17.6 (12.8–28.3)	20.6 (14.9–33.3)	22.1 (15.8–36.7)

Genotype 1b							
	Median per laboratory (IU/ml)*			Overall median (IU/ml)	CV % per laboratory* (95 % Confidence interval)		
GT1b 100 IU/ml							
CTM v2	158	169	171	166	18.9 (13.7–30.5)	24.1 (17.4–39.4)	32.3 (23.1–54.2)
HPS	198			198	17.7 (12.9–28.4)		
RealTi*m*e	87	96	123	97	15.7 (11.4–25.1)	18.8 (13.6–30.3)	19.4 (14.1–31.1)
GT1b 25 IU/ml							
CTM v2	28	29	40	31	41.9 (29.2–75.2)	50.3 (34.5–94.5)	54.9 (37.4–106.3)
HPS	34			34	42.5 (28.0–90.7)		
RealTi*m*e	17	19	38	22	16.2 (11.6–27.2)	27.7 (19.7–46.8)	34.9 (24.6–60.7)

* All values in increasing order independent of laboratory

In general, results obtained with CTM v2 and HPS when analysing the genotype 1b replicates at 25 IU/ml had higher coefficients of variation than corresponding genotype 1a results.

Median values for genotype 1a and 1b replicates at 100 and 25 IU/ml nominal concentrations across the participating laboratories are listed in Table 4. Compared to genotype 1b replicates, median values of genotype 1a replicates measured by HPS or CTM v2 showed discrepant quantification results relative to RealTi*m*e results (Table 4).

## Discussion

The results of this multicentre-per-assay study confirm and amend the conclusions of the previously reported initial investigation which was based on a one-laboratory-per-assay approach [7]. The intention of this study was to focus particularly on very low viremic specimens near the clinical cut-off of 25 IU/ml in a more comprehensive setting. RealTi*m*e demonstrated the highest detection rate (97 vs. 90 % for HPS and 88 % for CTM v2) in detecting WHO standard samples with nominal concentrations of 25 IU/ml and below. This corresponds to the ranking in the previous one-laboratory-per-assay study with standard dilutions at 25, 10 and 5 IU/ml. In the clinical sample analysis of genotypes 1a and 1b, intra-assay variation was consistently lower for RealTi*m*e (CVs at 100 and 25 IU/ml ranged from 13–21 % and 16–35 %, respectively) as compared to CTM v2 (15–32 and 28–55 %, respectively) and HPS (18–24 and 23–43 %, respectively), indicating a higher precision of this assay especially at a low nominal concentrations of 25 IU/ml. Despite the slightly different study design of the initial HCV LLV study with 10 replicates tested individually in 10 independent runs [7], results of this multicentric study are in line with the mean CV results obtained from the initial HCV LLV study (18 % at 100 IU/ml and 27 % at 25 IU/ml for RealTi*m*e, 32 and 53 % for CTM v2, and 32 and 48 % for HPS, respectively).

In registration trials for the new direct acting antivirals that were approved since 2014, SVR was consistently defined as HCV RNA < 25 IU/ml (either detected or undetected). Thus, a high precision at 25 IU/ml is of major importance to properly assess SVR. Furthermore, previously observed differences of quantification levels across assays were confirmed also in this multicentre-per-assay setting. This should be taken into consideration when switching assays during therapy and a comparison of quantification is suggested in this case.

Current guidelines of the European Association for the Study of the Liver (EASL) continue to recommend monitoring of viral load also in IFN-free regimens to assess patient adherence and therapy efficacy [8, 9]. Newer compounds and additional substances may lead to a simplification of virological management in HCV treatment in the future. However, it might also take several years for these compounds to become available and refundable especially in resource-limited countries.

## Conclusions

Presumably, quantification assays with high accuracy and precision will have their clinical relevance for the upcoming years since only one single measurement is used to assess SVR or to decide if a patient is eligible to continue an expensive antiviral therapy.
